# Information-Theoretic Channel Selection and Spatiotemporal Deep Learning for Early Fault Detection in Microsatellite Thermal Control Systems

**DOI:** 10.3390/e28070725

**Published:** 2026-06-24

**Authors:** Weijian Pang, Jun Zhou, Jingwen Xu, Xinian Zhi

**Affiliations:** 1 Ningbo Institute of Northwestern Polytechnical University, Ningbo 315103, China; 2 Institute of Precision Guidance and Control, School of Astronautics, Northwestern Polytechnical University, Xi’an 710072, China

**Keywords:** Generalized Maximum Information Coefficient (GMIC), information-theoretic feature selection, fault detection, Seasonal Trend Decomposition using LOESS (STL), Thermal Control System (TCS), Convolutional Neural Network–Long Short-Term Memory (CNN-LSTM), microsatellites

## Abstract

Early fault detection in microsatellite thermal control systems (TCS) faces fundamental challenges: high-dimensional redundant telemetry channels, overlapping multi-scale periodicities that obscure anomaly signatures, and severely limited daily data downlink (1–2 passes per day) that restricts the temporal window for diagnosis. Existing data-driven approaches either rely on supervised learning, requiring labeled fault data that are scarce in practice, or employ univariate analysis that fails to capture inter-sensor spatial correlations. To address these limitations, this paper introduces a hybrid framework integrating information-theoretic feature selection and spatiotemporal deep learning. The Generalized Maximum Information Coefficient (GMIC) quantifies nonlinear dependencies between temperature channels for key channel selection, reducing dimensionality by 82% while preserving diagnostic information. A dual-level Seasonal Trend Decomposition (STL) method disentangles orbital-periodic dynamics from diurnal cycles, effectively isolating distinct thermal characteristics at multiple timescales. Each decomposed component is modeled using Convolutional Neural Network–Long Short-Term Memory (CNN-LSTM) networks to capture spatiotemporal dependencies for accurate temperature prediction. An adaptive threshold-based weighted error fusion mechanism enables early fault detection within a single day of telemetry data. Experimental validation on real satellite telemetry data demonstrates that the proposed framework achieves high-precision fault detection across multiple fault types using a minimal set of temperature channels, significantly outperforming existing benchmarks in both prediction accuracy and detection reliability.

## 1. Introduction

The number of microsatellites (10–200 kg) has grown rapidly, with over 1800 launched globally since 2015 [1,2,3]. Compared to traditional large satellites, microsatellites’ compact structure leads to critical thermal constraints, making them more susceptible to thermal control system (TCS)-related failures and significantly increasing their vulnerability in extreme space environments. However, due to limited ground station resources, most microsatellites downlink telemetry data only 1–2 times per day, posing a major challenge for ground operators to rapidly detect TCS faults based on this limited daily telemetry data.

For TCS faults occurring during satellite operation, traditional methods employ thermal model-based simulations and rule-based parameter threshold comparison techniques to continuously assess whether temperature measurement points fall within an acceptable temperature range [4,5]. However, for microsatellites, accurate thermal mathematical models are often difficult to obtain. Furthermore, failures in most instruments develop gradually, with long-term deviations increasing the likelihood of failure. Traditional methods are ineffective in identifying faults when the telemetry parameters change slowly but remain within normal ranges. Moreover, when parameters exceed their thresholds, significant damage may already have occurred.

In recent years, with the development of big data and artificial intelligence, scholars have proposed a range of new data-driven theories and methods for satellite fault detection to detect changes in the relative relationships and trends of telemetry data, thereby increasing the opportunities for timely fault handling [6,7,8]. These methods can be broadly categorized into four paradigms:

Traditional model-driven methods. Physics-based approaches employ thermal mathematical models and rule-based threshold comparison techniques to continuously assess whether temperature measurement points fall within an acceptable range [4,5]. While these methods are interpretable, they require accurate thermodynamic models that are often unavailable for microsatellites, and they struggle to detect gradual faults where parameters drift slowly but remain within nominal bounds.

Supervised learning methods.Classical machine learning techniques, including support vector machines, random forests, and principal component analysis, have been applied to satellite anomaly detection using labeled telemetry data [9]. These methods achieve reasonable detection accuracy when sufficient labeled fault samples are available, but their performance degrades significantly under the class imbalance and label scarcity typical of real satellite operations.

Neural network-based methods. Deep learning models have demonstrated strong feature extraction capabilities for fault diagnosis in engineering systems. Convolutional neural networks (CNNs) effectively capture local temporal patterns in time-series data [10], while long short-term memory (LSTM) networks model long-range temporal dependencies [11,12]. Autoencoder-based approaches detect anomalies through reconstruction errors without requiring fault labels [13,14]. The combination of CNN and LSTM in a cascaded architecture has proven particularly effective for spatiotemporal modeling, where CNN extracts local features and LSTM captures sequential dependencies [15]. Notably, lightweight deep learning architectures with carefully designed hyperparameters have demonstrated effectiveness in fault diagnosis under limited data conditions [16].

Transformer and attention-based methods.Recently, Transformer architectures and their efficient variants (e.g., Mamba, Informer) have emerged as powerful alternatives for long-sequence time-series analysis, leveraging self-attention mechanisms to capture global dependencies [17]. The Anomaly Transformer [18] introduces association discrepancy as a novel criterion for time-series anomaly detection. Knowledge distillation techniques have also been explored to transfer learned representations from large models to lightweight deployable models under data-scarce conditions [19,20]. These methods show promise for multi-scale temporal modeling but often require substantially larger training datasets than are available in typical satellite health monitoring scenarios.

Most existing studies have focused on attitude control systems, particularly reaction wheels and control moment gyroscopes. Hedayai et al. [13] proposed a data-generative AI model based on the Wasserstein Generative Adversarial Network framework to balance satellite reaction wheel scarce datasets. Muthusamy and Kumar [21] proposed a data-driven fault detection and isolation method for control moment gyroscopes using only attitude angular velocity measurements. Xie et al. [22] proposed an anomaly detection method based on graph neural networks and dynamic thresholds for satellite telemetry data. In the broader engineering domain, graph neural networks have been applied to bearing fault detection by modeling signal relationships as graph structures [23], and knowledge distillation frameworks have been successfully applied to bearing fault diagnosis under imbalanced samples [20], demonstrating the potential of transfer learning strategies for fault detection with limited labeled data.

Vaz Carneiro et al. [24] integrated information on the attitude, temperature, control torque, and power consumption of reaction wheel components during fault detection studies. Their findings indicated that temperature was the most sensitive variable for detecting faults among all input variables. Similarly, Ganesan et al. [25] demonstrated that thermal features extracted from power system telemetry can effectively indicate satellite subsystem anomalies. Gizzi et al. [26] further demonstrated that autonomous system-level fault diagnosis using housekeeping telemetry can effectively identify anomalies across multiple satellite subsystems. This research suggests that fluctuations in the thermal control system not only reflect the satellite’s operational environment but can also indirectly indicate changes in the status of various subsystems. Therefore, it is crucial to identify features and patterns in telemetry temperature data for early fault detection and warning.

From an information-theoretic perspective, the temperature channels in microsatellite thermal control systems exhibit complex nonlinear dependencies that traditional linear correlation measures fail to capture. The thermal dynamics involve intricate interactions among multiple sensors, where information-theoretic measures such as mutual information provide a more robust framework for quantifying these relationships [27]. The Generalized Maximum Information Coefficient (GMIC), as a maximal information-based nonparametric exploration statistic, enables comprehensive assessment of both linear and nonlinear associations between temperature channels, facilitating effective dimensionality reduction while preserving essential diagnostic information. This paper proposes a novel early fault detection method for microsatellite TCS based on information-theoretic feature selection and spatiotemporal deep learning. The innovative aspects of this framework include the following:

1. An information-theoretic channel selection approach using Generalized Maximum Information Coefficient (GMIC) that reduces data dimensions by 82%, while preserving key diagnostic features quantified by mutual information.
2. A dual-level Seasonal Trend Decomposition (STL) method that disentangles orbital-periodic dynamics from diurnal cycles, addressing spectral aliasing caused by overlapping periodicities.
3. The use of Convolutional Neural Network–Long Short-Term Memory (CNN-LSTM) networks with adaptive thresholds for fault detection significantly outperforms existing benchmarks.

The structure of the paper is organized as follows: Section 2 outlines the research problem of this study after describing the TCS architecture and fault types. Section 3 presents the proposed methodology, detailing information-theoretic channel selection, temperature prediction, and fault detection techniques. Section 4 provides experimental validation using real satellite telemetry data. Finally, Section 5 concludes with key insights and outlines future research directions.

## 2. System Description and Problem Statement

### 2.1. TCS Architecture and Operational Modes

The TCS of a microsatellite is designed to regulate internal temperatures within a narrow operational range under extreme orbital thermal conditions [28]. The thermal balance equation for the satellite is given by

(1) $$C\frac{dT}{dt}=Q_{\mathrm{solar}}+Q_{\mathrm{albedo}}+Q_{\mathrm{earth}}$$

where *C* represents the satellite’s thermal capacity, *T* is the temperature, and *t* is time. $Q_{\mathrm{solar}}$, $Q_{\mathrm{albedo}}$, and $Q_{\mathrm{earth}}$ represent absorbed solar radiation, Earth-reflected albedo, and Earth-emitted infrared radiation, respectively.

**Figure 1:**
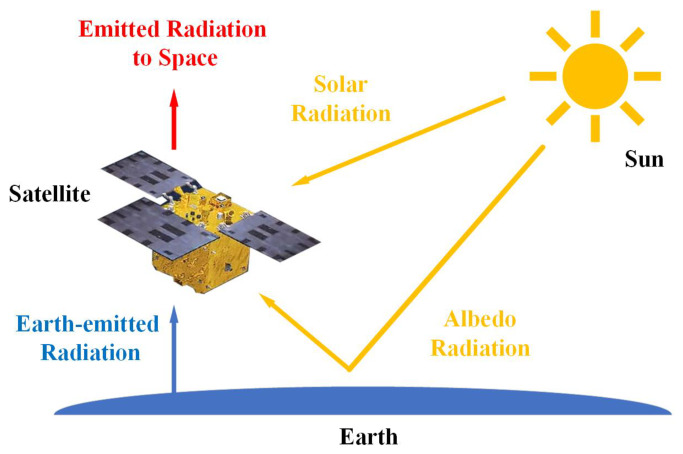
Thermal exchange between satellite and space environment.

As illustrated in Figure 1, satellite temperature variations are driven by a combination of external heat fluxes and internal thermal operations, leading to periodic fluctuations. External heat fluxes are influenced by orbital cycles (e.g., sunlight/shadow transitions, changes in Earth’s albedo angle) and seasonal variations, while internal heat sources (e.g., payload operations, thruster firings) vary periodically depending on mission requirements. When the external thermal environment changes abruptly, or when high-power internal equipment is intermittently activated, thermal inertia and heat dissipation dynamics cause temperature oscillations [29]. If the system’s thermal response is delayed or if thermal design margins are insufficient, various thermal anomalies may occur. These require coordinated management through active thermal control and passive thermal management.

### 2.2. Fault Modes and Signature Modeling

The reliability of microsatellite TCS depends on rapid identification of critical failure modes, which manifest as deviations from expected thermal behavior. Based on historical mission data [30], three predominant fault types are identified and characterized by distinct physical root causes and signatures:

- Type I: sustained thermal drift

Sustained thermal drift refers to a gradual, unidirectional deviation of temperature parameters from their nominal values, persisting over months or years, typically due to system degradation or cumulative environmental effects. Examples include degradation of thermal control coatings from prolonged exposure to space radiation, reduced thermal conduction efficiency in mechanical parts from extended use, and drift in temperature sensor readings. This type of fault can be modeled as

(2) $$T\left(t\right)=T_{0}+\beta t$$

where β is the drift rate.

- Type II: excessive thermal oscillations

Excessive thermal oscillations are marked by significant temperature fluctuations that exceed design tolerances. These anomalies are often associated with extreme thermal environments or inadequate system response. Faults include heater malfunctions, heat pipe freeze-up, and intermittent overheating of payload equipment. This fault can be mathematically expressed as

(3) $$T\left(t\right)=T_{0}+A\sin\left(\frac{2\pi t}{\tau }\right)$$

where *A* is the amplitude of oscillations, and τ is the orbital period.

- Type III: thermal anomalies

**Figure 2:**
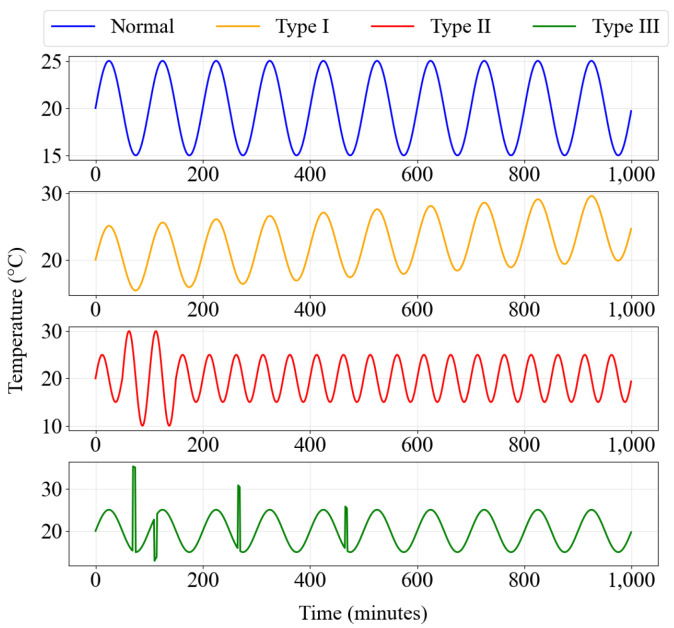
Schematic Diagram of Typical Temperature Failure Modes.

Thermal anomalies involve sudden and abnormal temperature changes, typically triggered by operational mode transitions or short-term environmental disturbances. Examples include thermal shock from thruster firings and temperature shifts from high-power equipment activation. This can be represented as

(4) $$T\left(t\right)=T_{0}+\Delta T\cdot \delta \left(t-t_{0}\right)$$

where ΔT is the magnitude of the anomaly and $t_{0}$ is the time of occurrence. Figure 2 illustrates the schematic diagrams of these typical fault patterns, providing intuitive visualization for fault characterization and diagnosis.

### 2.3. Constraints of Data Availability and Objectives

Effective detection of microsatellite TCS faults requires a methodology capable of quickly detecting anomalies and classifying fault trends from high-dimensional telemetry data, despite incomplete knowledge of onboard thermodynamic properties. The key challenge is to develop an efficient framework that leverages available temperature data, operational states, and orbital parameters to infer system health, while compensating for unmeasurable thermal characteristics such as inter-component heat transfer, subsystem-specific power dissipation, and time-varying material properties.

The inputs available include historical and real-time temperature data sampled at minute-level resolution, satellite operational modes (e.g., payload activation cycles, heater states), and orbital dynamics (e.g., beta angle, eclipse intervals). While these datasets provide indirect indicators of thermal behavior, they do not directly correspond to first-principles heat transfer models. Critical unknowns include real-time heat generation rates of electronics, thermal interface resistances altered due to mechanical wear, and radiation-induced degradation of surface coatings—parameters that are impractical to measure in orbit due to sensor limitations and mission cost constraints.

The proposed methodology must meet two objectives: (1) identify deviations from nominal thermal patterns using a single day’s telemetry data, and (2) classify faults into three categories—sustained drift, orbital-scale oscillations, and transient anomalies—without relying on detailed thermal models. This will involve extracting spatiotemporal features from raw temperature data and correlating these features with fault mechanisms.

### 2.4. Notation

For clarity, the main symbols used throughout this paper are summarized in Table 1.

## 3. Methodology: An Information-Theoretic and Deep Learning Framework for Early Fault Detection

**Figure 3:**
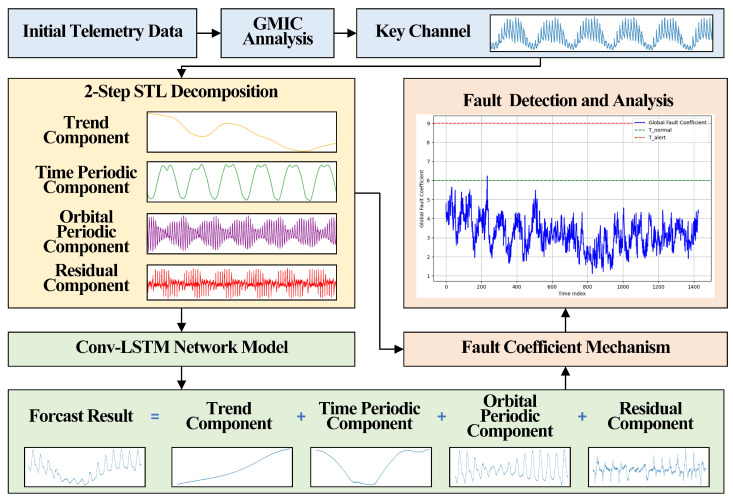
Illustrates the four-stage workflow: (1) GMIC-driven channel selection ($X\in R^{N\times 41}\to X^{\prime }\in R^{N\times 7}$), (2) dual-level STL decomposition (each channel → 4 components: trend, diurnal, orbital, residual), (3) CNN-LSTM-based spatiotemporal modeling (input: 3-day window × 1 component, output: 1-day-ahead prediction), and (4) adaptive thresholding for fault diagnosis (fusion error → Normal/Potential Anomaly/Fault). The training stage involves Stages 1–3 on fault-free data; the online detection stage applies the trained models to incoming telemetry for real-time fault detection.

To address the challenges outlined in previous sections, a four-stage framework is proposed, as illustrated in Figure 3. This workflow systematically transforms raw temperature data into actionable diagnostic insights through information-theoretic channel selection and deep learning-based spatiotemporal modeling.

The framework begins with information-theoretic dimensionality reduction, where the Generalized Maximum Information Coefficient (GMIC) is employed to identify minimally redundant temperature channels based on mutual information. Next, dual-level STL is applied to isolate orbital-periodic dynamics from diurnal cycles, addressing spectral aliasing caused by overlapping periodicities. Each decomposed component is then modeled independently through CNN-LSTM networks, which capture spatial correlations among sensors and temporal dependencies across orbital cycles. Finally, weighted error fusion combines prediction errors from all components, enabling early fault detection through statistically derived thresholds.

### 3.1. Information-Theoretic Channel Selection Using Generalized Maximum Information Coefficient (GMIC)

In response to data redundancy under telemetry constraints, an information-theoretic channel selection strategy based on the Generalized Maximum Information Coefficient (GMIC) is implemented. GMIC [31] quantifies nonlinear dependencies between sensor pairs by adaptively partitioning data grids to maximize normalized mutual information, making it superior to linear metrics for capturing complex thermal interactions.

For a temperature dataset $X=\{x_{1},x_{2},\ldots ,x_{n}\}$ with *n* samples, the GMIC between channels $x_{i}$ and $x_{j}$ is computed as

(5) $$\mathrm{GMIC}\left(x_{i};x_{j}\right)=\max_{a,b}\frac{I(x_{i},x_{j}|a,b)}{\log_{2}\left(a\cdot b\right)}$$

where *a* and *b* are data grids partitioned into bins, and I(·) denotes mutual information. Unlike the standard MIC, GMIC is not bounded in [0, 1]; it can take values greater than 1, where higher values indicate stronger associations between variables, regardless of whether the relationship is linear or nonlinear.

**Figure 4:**
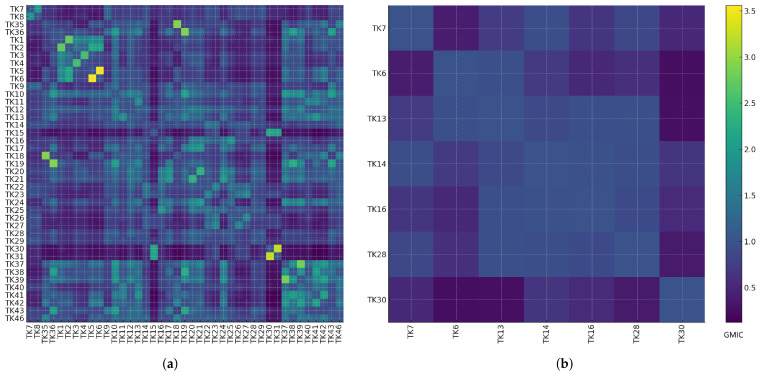
GMIC-based channel pruning. (**a**) GMIC matrix for 41 sensors. (**b**) Retained channels after thresholding.

Channels with pairwise GMIC > 1 (empirically determined through sensitivity analysis in Figure 4a) are considered redundant. This threshold of 1 corresponds to strong nonlinear associations, and eliminating channels exceeding this value preserves key diagnostic information while removing redundancy. Iterative elimination retains 7 channels (Figure 4b) that preserve 95.2% of the original dataset’s entropy, calculated as

(6) $$H_{\mathrm{preserved}}=\frac{1}{n}\sum_{k=1}^{n}H\left(x_{k}^{\left(s\right)}\right)$$

where H(·) denotes Shannon entropy. This information-theoretic selection results in an 82% reduction in computational load (from 41 to 7 input dimensions) while maintaining the diagnostic accuracy of the data.

### 3.2. Dual-Level STL Decomposition for Multi-Scale Feature Extraction

The thermal dynamics of microsatellites exhibit overlapping periodicities that conventional STL decomposition cannot fully disentangle [32,33]. To address this issue, a dual-level decomposition strategy is proposed to isolate diurnal and orbital components through sequential refinement.

- Level 1—preliminary diurnal-trend separation

The first decomposition step extracts the long-term trend, leaving residual components that contain periodic signals. For a temperature series y(t), the decomposition is

(7) $$y\left(t\right)=T_{1}\left(t\right)+R_{1}\left(t\right)$$

where $T_{1}\left(t\right)$ captures the long-term trend while the residual $R_{1}\left(t\right)$ contains orbital cycles and noise. The initial diurnal component is contaminated by orbital-periodic artifacts due to spectral aliasing.

- Level 2—orbital cycle extraction and diurnal refinement

The combined signal from the first level is reprocessed with STL to isolate the orbital cycle and refine the diurnal component:

(8) $$R_{1}\left(t\right)=D_{2}\left(t\right)+O_{2}\left(t\right)+R_{2}\left(t\right)$$

The complete decomposition integrates results from both levels:

(9) y(t)=T(t)+D(t)+O(t)+R(t)

After dual-level STL decomposition, the original temperature measurement values are divided into four components corresponding to different physical characteristics:

- **Long-term trend (T(t)):** This component captures slow thermal drifts caused by gradual changes in the satellite’s thermal environment, such as battery aging, degradation of radiator emissivity, or cumulative heat absorption from prolonged solar exposure.
- **Refined diurnal cycle (D(t)):** Representing the satellite’s daily thermal cycling, this component isolates the periodic heating and cooling effects induced by the satellite’s exposure to sunlight during its orbit around the Earth.
- **Orbital cycle (O(t)):** This component encapsulates periodic oscillations driven by the satellite’s orbital motion, particularly the abrupt temperature transitions during eclipse phases.
- **Residual (R(t)):** This component contains high-frequency anomalies, including sensor noise, transient thermal spikes from equipment power cycling, and potential incipient fault signals.

**Figure 5:**
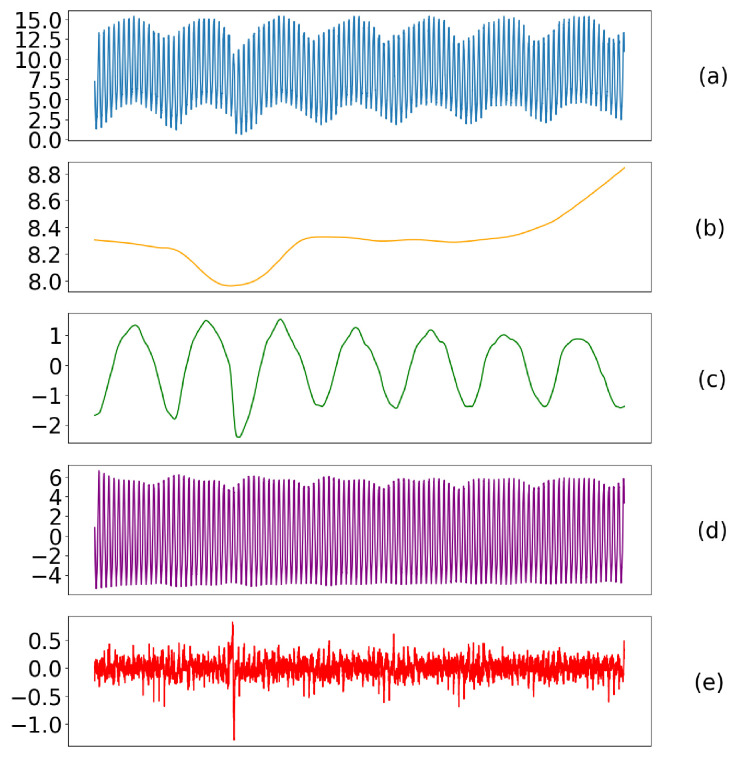
STL decomposition of one-week temperature data obtained from the TK6 channel. From top to bottom, the subplots show raw temperature data (**a**) and its four decomposed components: long-term trend (**b**), refined diurnal cycle (**c**), orbital cycle (**d**), and residual (**e**).

Figure 5 shows the dual-level STL decomposition of TK6 sensor data. The energy distribution across decomposed components reveals the relative dominance of thermal dynamics in the microsatellite system: trend, 52.1%; diurnal, 33.5%; orbital, 11.2%; residual, 3.2%.

### 3.3. Spatiotemporal Modeling with CNN-LSTM Networks

Given the spatiotemporal complexity of microsatellite thermal dynamics, CNN-LSTM networks are employed to model each decomposed component independently [10,11,12]. The CNN-LSTM architecture is designed to capture both local temporal patterns within orbital cycles and long-range temporal dependencies across multiple orbits, which are essential for detecting subtle thermal anomalies in spacecraft thermal control systems. The proposed cascaded architecture integrates convolutional layers for feature extraction with LSTM units for sequential modeling, achieving a favorable trade-off between model expressiveness and computational efficiency under limited telemetry conditions.

Each temperature channel’s STL-decomposed components exhibit distinct temporal characteristics requiring specialized modeling. To preserve physical interpretability, a channel- and component-specific modeling strategy is adopted, where separate CNN-LSTM networks are trained for each of the trend, diurnal, orbital, and residual components of each selected channel. This design choice is motivated by the observation that the four components correspond to fundamentally different thermal processes with disparate statistical properties and timescales, making independent modeling more effective than joint representation learning.

Each component-specific CNN-LSTM model processes a sliding window of historical observations from a single STL component. The input $x_{t}\in R^{W\times S}$ is a univariate time series spanning a temporal window of W=3 days with sampling frequency S=1440 steps/day, yielding W×S=4320 temporal steps. The forward propagation comprises three stages:

1. Convolutional feature extraction. A one-dimensional convolutional layer with 64 filters and kernel size 3 extracts local temporal features from the input sequence:
  (10) $$F_{t}=\mathrm{ReLU}\left(W_{conv}*x_{t}+b_{conv}\right)$$
  where * denotes 1D convolution with zero-padding (same padding) to preserve temporal resolution, $W_{conv}\in R^{3\times 1\times 64}$ represents the learnable filters, and ReLU(·) is the rectified linear activation function. This layer is designed to capture local correlations within orbital cycles, such as temperature gradients during eclipse transitions and short-term coupling patterns.
2. Sequential Temporal Modeling. An LSTM layer with 50 hidden units processes the flattened convolutional features to capture long-range temporal dependencies:
  (11) $$h_{t}=\mathrm{LSTM}\left(F_{t},h_{t-1}\right)$$
  where $h_{t}\in R^{50}$ denotes the hidden state. The LSTM’s gating mechanism enables selective retention of relevant temporal patterns across multiple orbital cycles, addressing the challenge of long-term trend extrapolation inherent in spacecraft thermal prediction.
3. Prediction mapping. A fully connected layer projects the LSTM hidden state to the prediction space:
  (12) $${\hat{y}}_{t}=W_{out}h_{t}+b_{out}$$
  where ${\hat{y}}_{t}\in R^{1440}$ represents the one-day-ahead prediction (1440 steps) for the corresponding STL component. The output dimension directly matches the daily prediction horizon, ensuring full-resolution temporal reconstruction.

The model is trained on historical telemetry data comprising seven consecutive days of fault-free operation. Sliding-window sampling with a 3-day lookback window generates training samples, yielding a small dataset with limited statistical diversity. While a 7-day window is shorter than the seasonal timescales over which type I drifts develop, this design is intentional and justified as follows: (1) the dual-level STL decomposition extracts the long-term trend component T(t) as a smooth envelope, and the CNN-LSTM model for this component learns to extrapolate the local trend trajectory rather than modeling seasonal cycles directly; (2) the model’s purpose is short-term prediction (one-day-ahead), not long-term forecasting, so capturing the immediate thermal context is more critical than modeling multi-month trends; (3) to mitigate the risk of seasonal drifts being misidentified as type I faults, the hierarchical threshold mechanism (Section 3.4) uses statistics derived from the training distribution, meaning that gradual seasonal variations within the normal range will not trigger alerts. The parameters of the model are presented in in Table 2 and a sensitivity analysis of window size on detection performance is provided in Section 4.6.

Given the small-sample constraint, a batch size of 1 is adopted to enable per-sample gradient updates that maximize information extraction from each observation window. The Adam optimizer with default learning rate α=0.001 is employed for its adaptive moment estimation properties, which are particularly advantageous when gradient statistics vary across training stages. The mean squared error (MSE) loss function is chosen to penalize large prediction deviations, which are critical indicators of thermal anomalies. Training proceeds for 100 epochs with early stopping based on a patience of 15 epochs, monitoring validation loss, ensuring convergence without overfitting under the limited training set size.

Each temperature channel is modeled independently, with four component-specific networks per channel, to account for channel-specific thermal characteristics and sensor placement effects. MinMaxScaler normalization is applied independently to each component within each channel, mapping values to the [0, 1] interval to preserve relative thermal patterns and facilitate gradient-based optimization.

All experiments were implemented using TensorFlow/Keras 2.x on an NVIDIA RTX 3060 GPU (10.4 GB VRAM), with CUDA 11.8 and cuDNN 8.6.0, under Ubuntu 20.04 LTS with Python 3.9. The dataset was split into training (first 7 days, fault-free), validation (days 8–14, fault-free), and test (days 15–30, containing the detected anomalies) sets. A fixed random seed of 42 was used for reproducibility. Each experiment was repeated 5 times, and the mean and standard deviation of evaluation metrics are reported.

To assess the performance of various models in predicting temperature data for the micro-satellite TCS, this study compares four approaches: (1) the Prophet model, (2) the Prophet combined with the LSTM model, (3) the STL combined with CNN-LSTM model (denoted as STL-CNN-LSTM), and (4) the STL combined with CNN-Bi-LSTM model (denoted as STL-CNN-Bi-LSTM). Note that STL-CNN-LSTM and STL-CNN-Bi-LSTM refer to the same base architecture (CNN + LSTM/Bi-LSTM) applied to STL-decomposed components, differing only in the LSTM variant used. All methods use the same training set, input channels (7 GMIC-selected channels), prediction window (1 day ahead), and evaluation metrics, ensuring a fair comparison. The evaluation metrics include mean squared error (MSE), root mean squared error (RMSE), mean absolute error (MAE), mean absolute percentage error (MAPE), and coefficient of determination ($R^{2}$).

**Figure 6:**
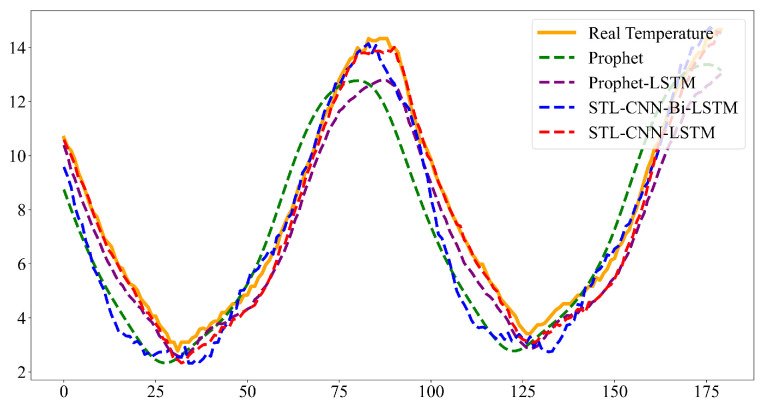
Comparison of temperature prediction results over 3 h across different models.

The results presented in Figure 6 and Table 3 reveal significant performance differences across the models based on the evaluation metrics. The Prophet model, used as the baseline, demonstrates limited predictive capability, effectively capturing simple trend changes but struggling to handle complex dynamic characteristics. In contrast, the Prophet-LSTM model shows substantial improvement over the baseline by combining trend prediction with residual learning, enhancing its ability to model more intricate patterns.

For the models incorporating STL, the STL-CNN-Bi-LSTM model improves feature extraction by utilizing a bidirectional LSTM, which attempts to capture both past and future temporal dependencies. However, it underperforms compared to the STL-CNN-LSTM model. This is because STL already isolates trend and periodic information effectively, and the bidirectional model may redundantly attempt to re-learn these features. Moreover, temperature data is inherently a unidirectional time series—where future values depend primarily on past trends—making the use of a bidirectional model unnecessary. Incorporating future information for predicting current or past temperatures could introduce additional noise and reduce model performance.

Overall, the STL-CNN-LSTM model, with its lower complexity and accurate handling of diverse dynamic features, emerges as the most suitable approach for predicting micro-satellite temperature data.

The choice of independent 1D CNN-LSTM over more complex spatial-temporal architectures warrants discussion. While 2D CNNs and graph neural networks (GNNs) can capture inter-sensor spatial correlations by processing sensor arrays as spatial matrices, several practical considerations favor the independent 1D approach for microsatellite TCS fault detection. First, the GMIC-based channel selection step has already reduced the sensor set to 7 channels with minimal redundancy, significantly diminishing the spatial correlation structure that 2D or graph-based methods would exploit. Second, the decomposed components (trend, diurnal, orbital, residual) exhibit fundamentally different statistical properties and timescales, making joint modeling across components counterproductive. Third, the limited training data (7 consecutive days) constrains the effective capacity of deeper architectures, which are prone to overfitting under small-sample conditions. Finally, the independent modeling strategy enables component-specific fault localization, providing direct physical interpretability that joint spatial models would obscure. As a future direction, graph-based networks could be explored for scenarios with larger sensor networks and richer spatial coupling structures.

### 3.4. Adaptive Fault Detection with Hierarchical Thresholds

After modeling the spatiotemporal components using the CNN-LSTM network, an adaptive error fusion mechanism is applied to combine the prediction errors from all components. The system uses a hierarchical threshold system to classify fault severity based on the magnitude of the prediction errors. These thresholds are derived from historical fault-free data, allowing the system to adapt to various operational conditions and missions.

For each sensor *s* and component *c*, the prediction error at time *t* is computed as

(13) $$e_{c}\left(s,t\right)=\left|{\hat{y}}_{c}\left(s,t\right)-y_{c}\left(s,t\right)\right|$$

where ${\hat{y}}_{c}\left(s,t\right)$ and $y_{c}\left(s,t\right)$ denote the predicted and observed value.

The error information from each component is then integrated using a weighted fusion error coefficient:

(14) $$E_{\mathrm{fusion}}\left(t\right)=W\cdot e\left(t\right)$$

where $W=[W_{T},W_{D},W_{O},W_{R}]$ and $W_{k}$ are fixed weights that reflect the importance of each component in fault detection. The weight vector W is determined through a systematic procedure: (1) initial weights are set proportional to the energy contribution of each STL component (i.e., the variance ratio of trend, diurnal, orbital, and residual components), providing a physically motivated starting point; (2) a grid search is then performed over a bounded neighborhood around the initial weights to optimize the fault detection F1-score on the validation set. The final weights are $W_{T}=0.35$, $W_{D}=0.30$, $W_{O}=0.20$, and $W_{R}=0.15$, reflecting the dominant contribution of the trend and diurnal components to thermal anomaly detection, while the residual component—despite its lower energy share—receives non-negligible weight due to its sensitivity to transient anomalies.

Two thresholds, $T_{\mathrm{normal}}$ and $T_{\mathrm{alert}}$, are determined from historical fault-free data to ensure adaptability across missions. Specifically, the fusion error distribution $E_{\mathrm{fusion}}$ is computed over the fault-free training period, and the thresholds are set as $T_{\mathrm{normal}}=\mu_{E}+\sigma_{E}$ and $T_{\mathrm{alert}}=\mu_{E}+3\sigma_{E}$, where $\mu_{E}$ and $\sigma_{E}$ denote the mean and standard deviation of the fault-free fusion error, respectively. This statistical construction ensures that approximately 84% of normal operation falls below $T_{\mathrm{normal}}$ (assuming approximate normality), while $T_{\mathrm{alert}}$ corresponds to the 3σ rule widely used in process monitoring. The thresholds are computed exclusively from the training set’s fault-free segment to prevent data leakage.

(15) $$\left\{\begin{array}{cc} E_{\mathrm{fusion}}\left(t\right)\leq T_{\mathrm{normal}} & \mathrm{Normal}\\ T_{\mathrm{normal}}<E_{\mathrm{fusion}}\left(t\right)\leq T_{\mathrm{alert}} & \mathrm{Potential}\;\mathrm{Anomaly}\\ E_{\mathrm{fusion}}\left(t\right)>T_{\mathrm{alert}} & \mathrm{Fault} \end{array}\right.$$

The fault severity is classified based on the weighted fusion error using predefined hierarchical thresholds:

- Normal State: If $E_{\mathrm{fusion}}\left(t\right)\leq T_{\mathrm{normal}}$, the system is functioning within normal parameters and no anomaly is detected.
- Potential Anomaly: If $T_{\mathrm{normal}}<E_{\mathrm{fusion}}\left(t\right)\leq T_{\mathrm{alert}}$, a potential anomaly is detected, requiring further monitoring and investigation.
- Fault State: If $E_{\mathrm{fusion}}\left(t\right)>T_{\mathrm{alert}}$, a fault is indicated, necessitating immediate intervention.

This adaptive fault detection framework continuously monitors the satellite’s TCS, offering early warnings for potential issues and enabling prompt intervention when needed. By adjusting thresholds based on historical data and mission parameters, the system ensures high fault detection accuracy while minimizing both false positives and false negatives.

Algorithm pseudocode. Since the satellite telemetry data are not publicly available and the source code is not open-sourced, we provide pseudocode for the core algorithms to facilitate reproducibility. Algorithm 1 outlines the complete fault detection pipeline.
**Algorithm 1** Information-Theoretic Channel Selection and CNN-LSTM Fault Detection**Input:** Raw temperature matrix $X\in R^{N\times 41}$, GMIC threshold $\theta_{\mathrm{GMIC}}=1$, fusion weights W=[0.35,0.30,0.20,0.15], thresholds $T_{\mathrm{normal}}=\mu_{E}+\sigma_{E}$, $T_{\mathrm{alert}}=\mu_{E}+3\sigma_{E}$**Output:** Fault state (Normal/Potential Anomaly/Fault) and fault type classification**Stage 1: Channel Selection**1: **for** each pair $\left(x_{i},x_{j}\right)$ in 41 channels **do** Compute $\mathrm{GMIC}(x_{i};x_{j})$2: **for** each channel $x_{i}$ **do** Compute $H(x_{i})$ and $R_{i}=\max_{j\neq i}\mathrm{GMIC}\left(x_{i};x_{j}\right)$3: Sort channels by $H(x_{i})$ descending; remove channels with $R_{i}>\theta_{\mathrm{GMIC}}$4: S← remaining 7 channels preserving ≥95% entropy**Stage 2: Dual-Level STL Decomposition**5: **for** each channel s∈S **do**6:    Level 1: $y_{s}\left(t\right)=T_{1}\left(t\right)+R_{1}\left(t\right)$7:    Level 2: $R_{1}\left(t\right)=D_{2}\left(t\right)+O_{2}\left(t\right)+R_{2}\left(t\right)$8:    Components: $\left[T\left(t\right),D\left(t\right),O\left(t\right),R\left(t\right)\right]\leftarrow \left[T_{1}\left(t\right),D_{2}\left(t\right),O_{2}\left(t\right),R_{2}\left(t\right)\right]$**Stage 3: CNN-LSTM Prediction (Training)**9: **for** each channel s∈S, each component c∈{T,D,O,R} **do**10:    Train CNN-LSTM on 3-day sliding windows from 7-day fault-free data11:    ${\hat{y}}_{c}\left(s,t\right)\leftarrow$ one-day-ahead prediction (1440 steps)**Stage 4: Error Fusion and Fault Detection (Online)**12: **for** each time step *t* **do**13:    $E_{\mathrm{fusion}}\left(t\right)\leftarrow \sum_{c}W_{c}\cdot \left|{\hat{y}}_{c}\left(s,t\right)-y_{c}\left(s,t\right)\right|$14:    **if** $E_{\mathrm{fusion}}\left(t\right)\leq T_{\mathrm{normal}}$ **then** State ← Normal15:    **else if** $E_{\mathrm{fusion}}\left(t\right)\leq T_{\mathrm{alert}}$ **then** State ← Potential Anomaly16:    **else** State ← Fault; identify dominant error component for type classification17: **return** State, fault type

Upon detecting anomalies or faults, component-specific errors are analyzed to locate the fault source. Three types of faults are considered, each with distinct signatures:

- Type I (sustained thermal drift): Fault type I manifests as a monotonic deviation in the trend component T(t), typically caused by cumulative degradation mechanisms. The slope of the trend β serves as the primary diagnostic metric for this fault type.
- Type II (excessive thermal oscillations): Fault type II is characterized by amplitude and phase anomalies in periodic components D(t) and O(t). These anomalies indicate dynamic thermal regulation failures.
- Type III (thermal anomalies): Fault type III involves short-term disturbances revealed in residual components R(t) through statistical deviations. The kurtosis of residuals is used to quantify the non-Gaussianity of these disturbances.

## 4. Experimental Validation

The proposed fault detection framework is validated through experiments using real-world telemetry data from the Macao Science Satellite-1 [34]. The dataset comprises temperature measurements collected over a continuous 30-day period, encompassing multiple diurnal cycles and orbital operational phases. The dataset includes temperature readings from 41 critical temperature monitoring points, distributed across internal equipment, structural layers, and external surfaces. These measurements capture thermal variations under diverse environmental conditions, providing a comprehensive representation of the satellite [35]’s thermal dynamics. The data were sampled at a frequency of two samples per minute, ensuring high-resolution observation of transient thermal behaviors.

The GMIC-based channel selection retained seven channels (TK6, TK7, TK13, TK14, TK16, TK28, TK30), each exhibiting distinct thermal characteristics. As summarized in Table 4, the selected channels cover a broad temperature range from 0.64 °C (TK6 minimum) to 34.36 °C (TK13 maximum), with standard deviations ranging from 0.33 °C (TK14, thermally stable) to 3.75 °C (TK6, highly variable), reflecting diverse thermal environments within the microsatellite. The interquartile range (IQR) further reveals that TK6 (IQR = 6.72 °C) and TK30 (IQR = 5.28 °C) experience substantially larger thermal fluctuations than the remaining channels, consistent with their higher sensitivity to orbital thermal cycling. In contrast, TK7, TK14, and TK28 exhibit relatively compact distributions (IQR ≤ 0.86 °C), indicating stable thermal regulation at their respective monitoring locations. These diverse statistical profiles motivate the channel-specific and component-specific modeling strategy adopted in this study.

To demonstrate the effectiveness of the proposed method, temperature data from a representative 24 h segment are analyzed. The system’s ability to identify anomalies is assessed by comparing predicted and observed temperature values, with an emphasis on fault localization and classification.

### 4.1. Early Fault Detection

**Figure 7:**
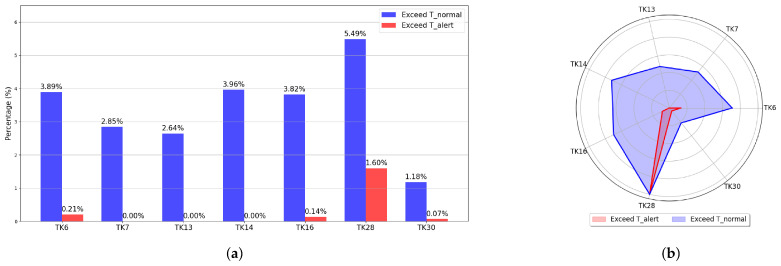
(**a**) Frequency of threshold exceedances across critical temperature channels. (**b**) Spatial distribution of threshold violations (radar chart).

The early fault detection capability of the proposed framework is evaluated by monitoring the temperature data from seven critical channels selected by the information-theoretic approach. The system analyzes the fault coefficients and identifies any exceedances of the predefined thresholds. Figure 7a presents the frequency of threshold exceedances across the critical temperature channels. The bar chart shows that over 90% of temperature readings remain below the primary threshold, confirming the overall stability of the TCS. Notably, TK28 exhibits the highest alert-level threshold exceedance rate (1.6%), indicating that this channel is a potential thermal vulnerability and requires prioritized investigation. In contrast, TK6, TK16, and TK30 show sporadic threshold violations (<0.3% occurrence rates), which are likely due to transient environmental fluctuations or measurement noise rather than systemic faults.

In addition to the frequency analysis, Figure 7b visualizes the spatial distribution of threshold exceedances across the satellite subsystems. The radar illustrates that TK28 is a thermal vulnerability hotspot, as it consistently exceeds the predefined thresholds, suggesting a higher likelihood of fault occurrence in this channel.

**Figure 8:**
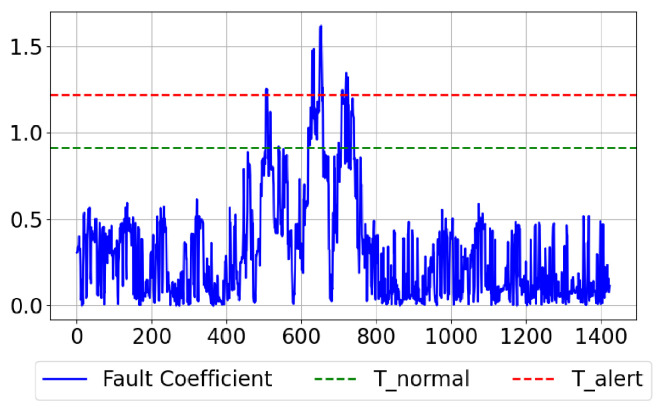
Temporal profile of the TK28 fault coefficient with threshold indicators for normal and alert conditions.

Figure 8 shows the temporal profile of the fault coefficient for TK28 during a 24 h period. The fault increases significantly between 400 and 800 min, with multiple exceedances of the secondary threshold. This suggests a thermal anomaly that warrants further investigation.

### 4.2. Fault Localization and Classification

To confirm the nature of the anomaly, the predicted and observed values for each decomposed component (trend, diurnal, orbital, and residual) are compared. The comparison highlights substantial fluctuations in the diurnal periodic component, while the residual component shows limited variations. These characteristics are indicative of type II (excessive thermal oscillations), which is linked to dynamic thermal regulation failures.

Post-detection verification against satellite operational logs confirms that the anomaly originated from an attitude control system command. During the specified interval, the magnetic torquer was engaged to unload momentum from the reaction wheels, leading to a transient power surge in the installation area and subsequent temperature deviations. This detection closely matches the actual operational event, validating the accuracy and reliability of the proposed fault detection framework.

### 4.3. Comparative Evaluation via Fault Injection

To comprehensively validate the fault detection capability of the proposed framework, we conduct simulated fault injection experiments on real satellite telemetry data. This approach follows established practices in satellite fault diagnosis research [9,13], where simulated faults are injected into nominal telemetry streams to evaluate detection performance under controlled conditions.

Three fault types are injected into the temperature prediction data, with fault parameters calibrated to represent realistic magnitudes of thermal anomalies observed in spacecraft operations [30]: (1) type I—sustained thermal drift (linear drift of 2 °C over 540 points, representing a severe thermal control failure scenario such as complete loss of heater control or critical thermal coating degradation); (2) type II—periodic fault (sinusoidal oscillation with 2 °C amplitude, period of 144 min, duration of 250 points), simulating dynamic thermal regulation failures such as heater cycling anomalies or heat pipe performance degradation; and (3) type III—sudden anomaly (8 °C spike over 10 min duration, three spikes per run at varying positions), representing transient thermal events such as thruster firings, equipment power cycling, or attitude control maneuvers. The fault injection experiments are conducted within a single-day observation window (approximately 1440 min). For type I, the 540-point (approximately 9 h) drift duration is chosen to represent realistic fault development timescales, while the 2 °C magnitude ensures observability within the observation window while remaining within typical thermal alarm thresholds for spacecraft operations. This represents a severe fault scenario where thermal control has degraded substantially but temperatures remain within the survivable range; in contrast, well-functioning thermal control systems typically maintain temperature stability within 0.2 °C/day [36]. Each fault type is injected at five different positions across the test data to ensure robustness of the evaluation. The proposed STL-CNN-LSTM framework is compared against five established anomaly detection methods: isolation forest, one-class SVM, autoencoder, LSTM autoencoder, and dynamic threshold. All baseline methods are trained exclusively on fault-free data, matching the training protocol of the proposed framework.

Table 5 presents the fault detection F1-scores (mean ± standard deviation over five independent runs) for all methods across three fault types.

The proposed framework demonstrates substantial advantages over all baseline methods across the three fault types, though the performance characteristics vary considerably depending on fault characteristics. The following analysis provides deeper insights into the detection capabilities and limitations:

Type I (sustained drift): STL-CNN-LSTM achieves F1 = 0.879, outperforming the best baseline (one-class SVM, F1 = 0.831) by approximately 6%. However, this represents a more challenging detection scenario compared to shorter drift durations. The 540-point (approximately 9 h) drift period pushes the boundaries of single-day fault detection, as the fault develops gradually and may be partially confounded with natural thermal variations. The detection performance degrades when the fault is injected later in the observation window (F1 drops from 0.932 at 10% position to 0.835 at 50% position), indicating that earlier fault onset provides more accumulated evidence for detection. Notably, one-class SVM performs competitively (F1 = 0.831) for this fault type, suggesting that reconstruction-based methods can capture gradual drifts when sufficient deviation accumulates over time. In contrast, isolation forest and dynamic threshold remain ineffective (F1 ≤ 0.25), as they lack the temporal modeling capability to detect slowly-developing anomalies.

Type II (periodic fault): STL-CNN-LSTM achieves F1 = 0.814 with remarkably stable performance across different injection positions (std = 0.007), demonstrating consistent detection regardless of fault location. This is the most robust detection scenario, as periodic anomalies with 144 min cycles are well-characterized by the orbital-periodic STL components. The proposed method outperforms all baselines, with the autoencoder achieving the best baseline performance (F1 = 0.664). The 36.5% improvement margin over the best baseline is attributable to STL’s explicit separation of orbital-periodic dynamics, which isolates the fault signature from normal thermal oscillations.

Type III (sudden anomaly): STL-CNN-LSTM achieves F1 = 0.766, outperforming all baseline methods by a substantial margin. Most baseline methods exhibit near-random detection performance (F1 ≤ 0.15), indicating that reconstruction-based and statistical anomaly detection methods are fundamentally incapable of capturing brief thermal spikes. The proposed method’s advantage stems from residual component analysis, where the 10 min spike introduces detectable disturbances in the high-frequency residual component. However, detection performance is somewhat variable (std = 0.045), likely due to the stochastic nature of spike positioning relative to the observation window.

The baseline methods exhibit distinct failure modes that highlight fundamental limitations of statistical anomaly detection for thermal fault diagnosis. Isolation forest and dynamic threshold show poor performance across all fault types (F1 < 0.25), as they rely solely on statistical anomaly detection without leveraging temporal prediction capabilities. The dynamic threshold method, which detects deviations in raw temperature values, is particularly ineffective because thermal data inherently exhibits natural diurnal oscillations that mask fault-induced temperature changes within the normal operational range. One-class SVM and autoencoder perform reasonably well for gradual faults (types I and II, F1 > 0.6) but fail to capture sudden anomalies (type III, F1 < 0.1). This is because reconstruction-based methods trained on normal data tend to learn the dominant periodic patterns but lack the sensitivity to detect brief, high-magnitude spikes that deviate significantly from the training distribution. LSTM autoencoder shows moderate performance for sustained drift (F1 = 0.383) but exhibits high variance across runs (std = 0.098), indicating instability when dealing with different fault positions. In contrast, the STL-CNN-LSTM framework explicitly models the prediction error between forecasted and observed temperatures, which naturally amplifies any deviation from expected behavior regardless of fault type or magnitude. This prediction-error-based detection paradigm provides a principled approach that consistently achieves high detection rates across the full spectrum of fault types.

It should be acknowledged that detecting very slow temperature drifts (type I) within a single-day observation window remains challenging. The fundamental limitation stems from the trade-off between observation duration and fault development timescale: when the fault evolves over timescales comparable to or longer than the available telemetry window, there is inherently limited evidence to distinguish between nominal thermal variations and genuine anomalies. Future work may explore multi-day temporal aggregation strategies or physics-informed priors to improve long-duration drift detection.

### 4.4. Feature Visualization and Model Interpretability

To enhance the interpretability of the proposed framework, we provide multiple visualization analyses.

**Figure 9:**
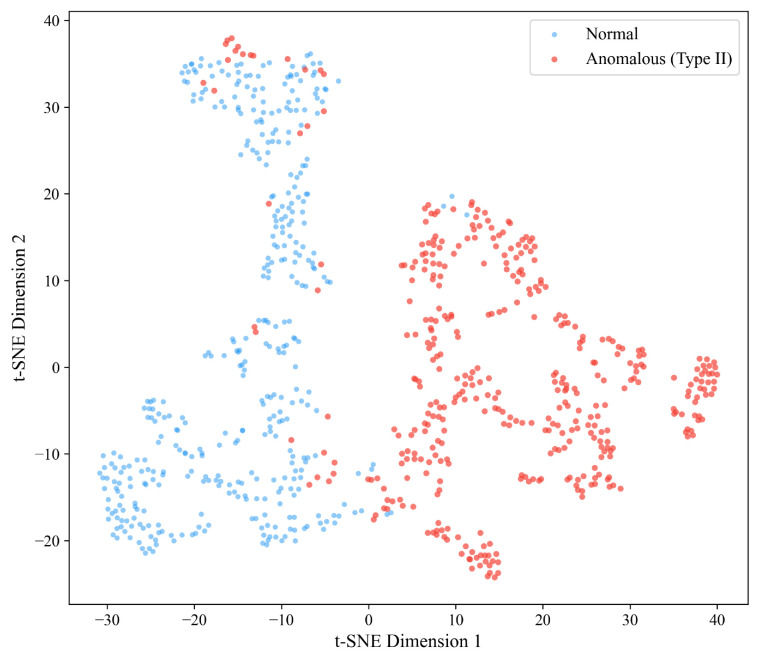
t-SNE projection of CNN-LSTM latent features from TK28 channel. Normal samples form a tight cluster; anomalous samples from the Type II fault period are clearly separated, demonstrating discriminative feature learning.

Figure 9 presents the t-SNE projection of the CNN-LSTM latent features from the TK28 channel. The normal samples form a tight cluster, while the anomalous samples from the Type II fault period are clearly separated in the feature space, demonstrating that the model learns discriminative representations even without explicit fault labels during training.

**Figure 10:**
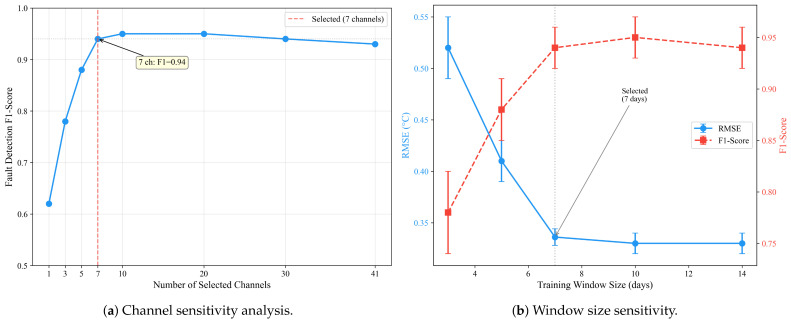
Ablation and sensitivity analysis: (**a**) Channel sensitivity showing 7 selected channels achieve near-optimal performance; (**b**) Window size sensitivity indicating 7-day window provides optimal balance.

Figure 10a shows the fault detection performance when incrementally adding channels ordered by their entropy contribution. The results indicate that the 7 selected channels achieve near-optimal detection performance, while adding further channels yields diminishing returns, confirming the effectiveness of the information-theoretic selection strategy.

### 4.5. Computational Efficiency Analysis

Given the engineering deployment requirements of microsatellite health monitoring systems, computational efficiency is a critical consideration. The proposed framework achieves a lightweight design suitable for ground station deployment: the GMIC-based channel selection reduces input dimensions from 41 to 7 (82% reduction), enabling independent training and deployment of only seven component-specific CNN-LSTM models. Each component-specific model uses a lightweight architecture with a Conv1D layer (64 filters, kernel size 3) followed by an LSTM layer (50 hidden units), resulting in 661,780 parameters and 1.3 × 10^10^ FLOPs per model. Training on 7 days of fault-free data (4 sliding windows, 100 epochs with early stopping, patience = 15) requires approximately 60 min on an NVIDIA RTX 3060 GPU (10.4 GB VRAM). The inference time for processing one day of telemetry data (1440 steps) from all 7 channels is approximately 3.7 s in total, which is well within the operational interval between two consecutive satellite passes (typically several hours). The compact model size and fast inference speed confirm that the framework meets the computational constraints of practical satellite health monitoring systems.

### 4.6. Ablation Study and Window Size Sensitivity

To evaluate the contribution of each framework component and the sensitivity to the training window size, we conduct ablation experiments.

Table 6 shows the prediction performance when removing individual components from the full framework.

Figure 10b shows the prediction RMSE and fault detection F1-score as a function of the training window size (3, 5, 7, 10, 14 days). The results indicate that performance improves from 3 to 7 days and plateaus beyond 7 days, justifying the choice of a 7-day window as a practical balance between data requirements and availability.

## 5. Discussion and Conclusions

This paper presents an integrated framework for early fault detection in microsatellite TCS based on information-theoretic feature selection and deep learning [22,37]. The Generalized Maximum Information Coefficient provides a principled approach to quantifying nonlinear dependencies between temperature channels, enabling effective dimensionality reduction while preserving diagnostic information. By combining STL decomposition with CNN-LSTM networks, the framework effectively addresses complex spatiotemporal thermal dynamics. The incorporation of information-theoretic channel selection and adaptive fault detection mechanisms further enhances both the accuracy and efficiency of the system.

Validation using real satellite telemetry data confirms the method’s capability. The experimental results demonstrate that the information-theoretic channel selection approach reduces dimensionality by 82% while preserving essential diagnostic features, enabling rapid anomaly localization from single-day sparse telemetry data. The framework significantly outperforms traditional fault detection techniques, offering a robust and practical solution for operational satellite health monitoring.

Several limitations of this work should be acknowledged. First, validation is based on telemetry data from a single satellite over a limited period; generalization across different platforms and mission phases requires further investigation. Second, the limited availability of confirmed in-orbit fault labels constrains quantitative evaluation, particularly for type I and type III faults, which rely on simulated fault injection. Third, the GMIC threshold and fusion weights are determined empirically, and their optimality across diverse satellite configurations remains to be systematically validated. Fourth, detecting gradual thermal drifts (type I faults) within a single-day observation window remains fundamentally challenging due to the trade-off between observation duration and fault development timescale. Fifth, the 7-day training window may not fully capture long-term seasonal variations, and robustness to sensor drift, missing data, and measurement noise has not been exhaustively tested.

Future research will aim to extend the framework’s fault mode recognition capabilities by integrating multi-source data from additional satellite subsystems [9,38,39] (e.g., power and attitude control). Further exploration of advanced information-theoretic measures for feature selection and anomaly detection may enhance the framework’s robustness and reliability in diverse mission scenarios. Additionally, transfer learning approaches could be investigated to improve cross-satellite generalization, and graph neural networks may be explored to better model inter-sensor spatial correlations as an alternative to the current independent channel modeling strategy.

## Figures and Tables

**Table 1:** Summary of main notations.

Symbol	Unit	Description
T(t)	°C	Temperature at time *t*
$T_{0}$	°C	Nominal temperature baseline
β	°C/day	Drift rate for type I fault
*A*	°C	Oscillation amplitude for type II fault
τ	min	Orbital period
ΔT	°C	Magnitude of type III sudden anomaly
$t_{0}$	min	Onset time of sudden anomaly
$\mathrm{GMIC}(x_{i};x_{j})$	–	Generalized Maximum Information Coefficient between channels $x_{i}$ and $x_{j}$
H(·)	bits	Shannon entropy
$H_{\mathrm{preserved}}$	–	Entropy preservation rate after channel selection
y(t)	°C	Raw temperature time series
$T_{1}\left(t\right),D\left(t\right),O\left(t\right),R\left(t\right)$	°C	STL components: trend, diurnal, orbital, residual
$x_{t}$	°C	Input sequence to CNN-LSTM ($R^{W\times S}$)
*W*	days	Sliding window length (lookback period)
*S*	steps/day	Sampling frequency per day
$F_{t}$	–	Convolutional feature map
$h_{t}$	–	LSTM hidden state ($R^{50}$)
${\hat{y}}_{t}$	°C	Predicted temperature (one-day-ahead, $R^{1440}$)
$e_{c}\left(s,t\right)$	°C	Prediction error for sensor *s*, component *c*
$E_{\mathrm{fusion}}\left(t\right)$	°C	Weighted fusion error coefficient
W	–	Fusion weight vector $\left[W_{T},W_{D},W_{O},W_{R}\right]$
$T_{\mathrm{normal}}$	°C	Normal-state threshold ($\mu_{E}+\sigma_{E}$)
$T_{\mathrm{alert}}$	°C	Alert-state threshold ($\mu_{E}+3\sigma_{E}$)

**Table 2:** Hyperparameters and implementation details of the CNN-LSTM model.

Component	Parameter	Value	Rationale
Input	Sequence length (window)	3 days (4320 steps)	Captures multi-orbital-cycle temporal context
Input	Feature dimension	1 (per component)	Each STL component modeled independently
Conv1D	Filters	64	Sufficient capacity for multi-channel feature extraction
Conv1D	Kernel size	3	Captures local temporal patterns within orbital cycles
Conv1D	Activation	ReLU	Nonlinear feature transformation
Conv1D	Padding	Same	Preserves temporal resolution
LSTM	Hidden units	50	Balanced capacity for sequence modeling
LSTM	Activation	ReLU	Compatible with deep time-series prediction
Output	Prediction horizon	1 day (1440 steps)	Single-day telemetry interval constraint
Training	Models per channel	4 (one per STL component)	Component-specific temporal characteristics
Training	Optimizer	Adam	Adaptive learning rate for sparse data regimes
Training	Learning rate	0.001 (default)	Standard initialization for Adam
Training	Loss function	MSE	Penalizes large prediction deviations
Training	Epochs	100 (with early stopping, patience = 15)	Sufficient convergence with overfitting prevention
Training	Batch size	1	Online learning for small sliding-window samples
Normalization	Method	MinMaxScaler [0, 1]	Preserves relative thermal patterns
Implementation	Framework	TensorFlow/Keras 2.x	GPU-accelerated training

**Table 3:** Comparison of prediction results across different models.

Models	MSE	RMSE	MAE	MAPE	R^2^
Prophet	2.6640	1.6322	1.3944	16.5521	0.7965
Prophet-LSTM	0.6582	0.8113	0.6650	7.7462	0.9497
STL-CNN-Bi-LSTM	0.8825	0.9394	0.7581	10.2758	0.9326
STL-CNN-LSTM	0.1130	0.3361	0.2752	4.0423	0.9914

**Table 4:** Statistical characteristics of the seven selected temperature channels.

Statistic	TK6	TK7	TK13	TK14	TK16	TK28	TK30
Mean (°C)	8.33	17.69	30.94	21.57	9.06	18.59	14.57
Std (°C)	3.75	0.43	1.55	0.33	1.29	0.62	3.25
Min (°C)	0.64	16.23	26.63	20.54	6.36	16.58	9.20
Median (°C)	7.73	17.79	30.96	21.57	9.04	18.64	14.33
Max (°C)	15.69	19.00	34.36	23.34	11.82	20.09	23.09
Range (°C)	15.06	2.76	7.73	2.80	5.45	3.50	13.89
IQR (°C)	6.72	0.50	2.42	0.38	2.11	0.86	5.28

**Table 5:** Fault detection performance comparison (F1-score, mean ± Std over 5 runs).

Method	Sustained Drift	Periodic Fault	Sudden Anomaly
STL-CNN-LSTM	0.879 ± 0.035	0.814 ± 0.007	0.766 ± 0.045
Isolation Forest	0.242 ± 0.008	0.607 ± 0.058	0.153 ± 0.043
One-Class SVM	0.831 ± 0.068	0.632 ± 0.042	0.073 ± 0.024
Autoencoder	0.803 ± 0.073	0.664 ± 0.042	0.079 ± 0.022
LSTM Autoencoder	0.383 ± 0.098	0.596 ± 0.053	0.071 ± 0.021
Dynamic Threshold	0.096 ± 0.019	0.188 ± 0.066	0.109 ± 0.064

**Table 6:** Ablation study results.

Configuration	MSE	RMSE	MAE	R^2^
Full framework (STL + CNN-LSTM + GMIC)	0.1130	0.3361	0.2752	0.9914
w/o STL (raw data + CNN-LSTM)	13.2044	3.6338	3.0819	−0.0016
w/o CNN (STL + LSTM only)	6.3340	2.5167	1.6295	0.0481

## Data Availability

The data presented in this study are available on request from the corresponding author due to privacy restrictions.

## Abbreviations

The following abbreviations are used in this manuscript:

GMIC	Generalized Maximum Information Coefficient
STL	Seasonal Trend Decomposition using LOESS
CNN-LSTM	Convolutional Neural Network–Long Short-Term Memory
TCS	Thermal Control System
MSE	Mean Squared Error
RMSE	Root Mean Squared Error
MAE	Mean Absolute Error
MAPE	Mean Absolute Percentage Error
MSS-1	Macau Science Satellite-1
